# The Outcome of Laparoscopic Cholecystectomy in Pregnant Women

**DOI:** 10.7759/cureus.80005

**Published:** 2025-03-03

**Authors:** Vinod Kumar Singhal, Faris Dawood Alaswad, Nufra Senofer, Varsha Ojha, Adil Md Suleman

**Affiliations:** 1 General Surgery, Prime Hospital, Dubai, ARE; 2 Surgery, Gladstone Hospital, Queensland, AUS; 3 ENT, Prime Hospital, Dubai, ARE; 4 Obstetrics and Gynecology, Prime Hospital, Dubai, ARE

**Keywords:** gallbladder stone, laparoscopic cholecystectomy, post operative management, pregnancy, surgical management

## Abstract

Objective: This study aims to critically evaluate the safety, feasibility, and clinical outcomes of laparoscopic cholecystectomy (LC) in pregnant women.

Methodology: A retrospective observational study was conducted, reviewing the medical records of 56 pregnant women who underwent LC for gallbladder stones at Prime Hospital, UAE, between January 2015 and December 2023. The inclusion criteria included pregnant women aged 18-42 years who underwent LC for acute or chronic cholecystitis, biliary colic, or in the immediate postpartum period. Exclusion criteria encompassed non-surgical cases and incomplete records. Diagnoses were based on clinical and imaging findings, and all surgeries adhered to a strict protocol to minimize preterm delivery risks. Data on demographics, operative details, and outcomes were analyzed using SPSS (IBM Corp., Armonk, NY).

Results: Among the 56 cases of LC, the participants had a mean age of 32.5 years and an average body mass index (BMI) of 28.4 kg/m², with a mean gestational age of 22.7 weeks. The majority of participants were multiparous (34, 60.7%). Comorbid conditions such as diabetes and hypertension were observed in 12 (21.4%) and 8 (14.3%) cases, respectively. The primary surgical indications included symptomatic cholelithiasis (30, 53.6%) and cholecystitis (20, 35.7%). Intraoperative complications were rare, with minimal bleeding (3, 5.4%) and a low conversion rate to open surgery (2, 3.6%). Postoperative pain was the most common complication (40, 71.4%). Obstetric outcomes included preterm labor (4, 7.1%) and fetal distress (3, 5.4%), though neonatal outcomes were favorable, with high APGAR scores and no fetal deaths reported.

Conclusions: LC in pregnant women is a safe and feasible procedure characterized by low rates of intraoperative and postoperative complications. Postoperative pain was the most frequently observed issue. The majority of deliveries were full-term, with favorable neonatal outcomes. These findings support LC as a viable treatment for gallbladder disease during pregnancy, mainly when performed in the second trimester.

## Introduction

Pregnancy induces a complex interplay of hormonal and physiological changes that affect various organ systems, including the biliary system. One of the most common conditions associated with the biliary tract in pregnant women is gallbladder disease, characterized by the formation of gallstones, bile sludge, and potentially symptomatic cholecystitis [1]. The prevalence of gallstones in pregnant women ranges from 5% to 12%, with symptomatic biliary disease reported in 0.05% to 3% of cases [2]. These disorders result from hormonal fluctuations during pregnancy, which alter the balance of bile components and contribute to the development of cholelithiasis. Increased cholesterol, reduced bile acid, and delayed gallbladder emptying collectively lead to bile stasis and supersaturation, promoting gallstone formation [1].

Elevated levels of reproductive hormones increase the cholesterol saturation of bile and impair the gallbladder’s response to cholecystokinin, a hormone essential for gallbladder contraction. Furthermore, increased cholic acid, decreased chenodeoxycholic acid, impaired enterohepatic circulation, and enhanced bile stasis collectively contribute to the elevated risk of gallstone formation during pregnancy [3]. Additional risk factors, including obesity, elevated body mass index (BMI), and multiparity, further predispose pregnant women to cholelithiasis [4,5]. Overweight and obesity, in particular, lead to higher cholesterol concentrations in bile, significantly increasing the likelihood of gallstone formation [5].

The management of gallstone disease during pregnancy poses unique challenges, as the health of both the mother and fetus must be carefully prioritized. Initial conservative treatment typically includes hospitalization, intravenous fluid administration, bowel rest, analgesics, and antibiotics [6]. However, approximately 40% of patients do not respond to medical management, making surgical intervention necessary. Cholecystectomy is the second most common non-obstetric surgical procedure performed during pregnancy [7]. While conservative approaches may provide temporary relief, delayed or inadequate treatment often results in recurrent symptoms and significant maternal morbidity. As a result, surgical removal of gallstones is frequently regarded as the definitive treatment [8].

Traditionally, open cholecystectomy was the preferred approach for managing symptomatic gallstones during pregnancy. However, recent advancements in laparoscopic techniques have demonstrated the safety and feasibility of laparoscopic cholecystectomy (LC) in pregnant women [9]. The laparoscopic approach provides several advantages, including shorter hospital stays, fewer postoperative complications, lower financial costs, reduced maternal morbidity and mortality, and fewer fetal complications [10]. Despite these benefits, concerns persist regarding its safety, particularly during the first trimester, when the risk of fetal loss is highest. Emerging evidence suggests that performing LC during the second trimester minimizes both fetal and maternal risks, making it a safer alternative to conservative management and open surgery [11].

Several studies have evaluated the outcomes of gallstone disease during pregnancy, emphasizing the benefits of surgical management via LC. Research indicates that LC is associated with a lower risk of readmission compared to conservative treatment [12]. However, significant gaps remain in the current data, particularly regarding the incidence and trends of surgical management for gallstone disease during pregnancy. This gap underscores the need for further research to understand these trends and outcomes better. Therefore, this study aims to critically evaluate the safety, feasibility, and clinical outcomes of LC in pregnant women.

## Materials and methods

Study design

This study was a retrospective observational analysis of pregnant patients who underwent LC at Prime Hospital, UAE, between January 2015 and December 2023. The medical records of all eligible patients were reviewed to assess the safety and outcomes of the procedure during pregnancy. A total of 56 pregnant women diagnosed with gallbladder stones were included in the study. The decision to proceed with surgery was made jointly by the attending surgeon and the patient, considering the risks and benefits of the intervention. Ethical approval was obtained from the hospital’s ethics committee, and informed consent was acquired from all participants before data collection.

Inclusion and exclusion criteria

The study included pregnant women between the ages of 18 and 42 who underwent LC during any trimester of pregnancy. Patients with acute cholecystitis, chronic cholecystitis, or symptomatic biliary colic that did not resolve with conservative management were eligible for inclusion. Women with asymptomatic gallstones who did not require surgical intervention were excluded from the study. Additionally, patients with incomplete medical records or unconfirmed diagnoses of acute cholecystitis were not included to ensure the accuracy and reliability of the findings.

Gestational age distribution

Among the 56 patients in the study, 22 (39.2%) were in the first trimester, 26 (46.4%) were in the second trimester, and eight (14.2%) were in the third trimester at the time of surgery. The timing of surgical intervention was based on the severity of symptoms, risk of complications, and overall maternal and fetal well-being.

Diagnosis

The diagnosis of uncomplicated gallstones was based on the presence of biliary colic without systemic inflammation. These patients exhibited a negative Murphy’s sign, as well as expected laboratory results and ultrasound findings that confirmed the presence of gallstones or biliary sludge without gallbladder wall thickening or common bile duct dilation. Biliary pancreatitis was diagnosed in patients who presented with epigastric pain radiating to the back, nausea, and vomiting, with confirmation through elevated serum amylase levels that were at least three times the standard limit or by imaging findings suggestive of pancreatitis. Acute cholecystitis was diagnosed in patients with right upper quadrant pain, a positive Murphy’s sign, elevated inflammatory markers, and ultrasound findings such as gallbladder wall thickening, edema, or pericholecystic fluid. The American Society of Anesthesiologists (ASA) classification system was used to categorize pregnant patients based on their preoperative health status and associated surgical risks [13].

Definitions

Preterm birth was defined as delivery occurring between 22 and 36 weeks and six days of gestation, while spontaneous abortion referred to pregnancy loss before 20 weeks or when the fetal weight was less than 500 g. The gestational period was categorized into trimesters, with the first trimester extending up to 13 weeks and six days, the second trimester from 14 to 27 weeks and six days, and the third trimester from 28 weeks onward. To minimize the risk of preterm labor, a close clinical surveillance protocol was followed, which included continuous maternal and fetal monitoring, obstetric evaluation 24 hours before and after surgery, and cardiotocographic recordings to assess fetal well-being.

Preoperative and surgical management

Preoperative Management

Before surgery, all patients received conservative treatment, including broad-spectrum antibiotics such as cefotaxime and metronidazole, intravenous fluids, and spasmolytics for pain management. The surgical and obstetric teams monitored patients through regular assessments of temperature, vital signs, and abdominal examinations. Obstetric ultrasonography was performed to evaluate fetal health and ensure that there were no contraindications to proceeding with surgery.

Surgical Technique

LC was performed under general anesthesia with careful modifications to minimize risks for both the mother and the fetus. The first trocar was inserted using the open (Hasson) technique to prevent injury, and the uterus was palpated before trocar placement to determine its position. The pneumoperitoneum was maintained at 10 to 12 mmHg to reduce intra-abdominal pressure and minimize fetal stress. The standard four-port laparoscopic technique was used, with careful dissection to reduce tissue trauma. Electrocautery and surgical clips were used for hemostasis and bile duct closure. Intraoperative cholangiography was performed selectively in cases where choledocholithiasis was suspected. Obstetric ultrasonography was repeated postoperatively to assess fetal well-being and detect potential complications. Postoperative care included close maternal and fetal monitoring, early mobilization, and gradual resumption of oral intake to promote recovery and reduce postoperative risks.

Data collection

Data were collected retrospectively through a detailed review of electronic medical records and organized systematically for analysis. Patient demographics, including age, body mass index (BMI), smoking status, diabetes status, and gestational age at surgery, were recorded. Operative details such as surgical indication, duration of surgery, time from decision to procedure, use of abdominal shields, presence of choledocholithiasis, and intraoperative spillage were also documented. Postoperative outcomes, including surgical and obstetric complications, neonatal health, mode of delivery, and Apgar scores, were collected to assess the impact of LC on pregnancy outcomes.

Statistical analysis

Data analysis was performed using SPSS version 26 (IBM Corp., Armonk, NY). Descriptive statistics were calculated to summarize patient demographics, operative data, and postoperative outcomes. Continuous variables were presented as mean values with standard deviation, while categorical variables were expressed as frequencies and percentages. Logistic regression analysis was conducted to identify factors associated with postoperative complications, including patient age, BMI, gestational age, parity, and ASA scores. Results were reported with coefficients, standard errors, p-values, and 95% confidence intervals. A p-value of less than 0.05 was considered statistically significant. The findings were presented in tables and graphs for clarity and ease of interpretation.

## Results

The mean age was 32.5 ± 4.2 years, while the mean BMI was 28.4 ± 3.1 kg/m². The mean gestational age was 22.7 ± 5.4 weeks. Regarding parity, 22 (39.3%) participants were nulliparous, and 34 (60.7%) participants were multiparous. Among comorbidities, 12 (21.4%) participants had diabetes, and 8 (14.3%) participants had hypertension (Table 1).

**Table 1:** Demographic and clinical characteristics of the study population (n = 56).

Variables	Total number, *n* (%)
Mean age (years)	32.5 ± 4.2
Mean body mass index (kg/m^2^)	28.4 ± 3.1
Mean gestational age (weeks)	22.7 ± 5.4
Parity	
Nulliparous	22 (39.3%)
Multiparous	34 (60.7%)
Comorbidities	
Diabetic	12 (21.4%)
Hypertension	8 (14.3%)

Among the symptoms, pain was the most common, reported by 48 (85.7%) participants, followed by nausea or vomiting, experienced by 36 (64.3%) participants, and fever, reported by 10 (17.9%) participants. Regarding the indications for surgery, 30 (53.6%) participants underwent surgery due to symptomatic cholelithiasis, 20 (35.7%) due to cholecystitis, and 6 (10.7%) for biliary colic. The distribution of ASA scores revealed that 14 (25%) participants were classified as Class I, 30 (53.6%) as Class II, 10 (17.9%) as Class III, and 2 (3.6%) as Class IV. These data provide detailed insights into the clinical presentation and surgical risk profiles of the participants (Table 2).

**Table 2:** Preoperative symptoms and surgical indications. ASA, American Society of Anesthesiologists

Variables	Total number, *n* (%)
Symptoms	
Pain	48 (85.7%)
Nausea/Vomiting	36 (64.3%)
Fever	10 (17.9%)
Indication of Surgery	
Symptomatic cholelithiasis	30 (53.6%)
Cholecystitis	20 (35.7 %)
Biliary colic	6 (10.7%)
ASA score	
Class I	14 (25%)
Class II	30 (53.6%)
Class III	10 (17.9%)
Class IV	2 (3.6%)

Table 3 summarizes the mean surgery time and intraoperative complications observed among the study participants. The mean surgery time was 75 ± 15 minutes. Regarding intraoperative complications, bleeding was reported in 3 (5.4%) participants, injury to surrounding organs occurred in 1 (1.8%) participant, and conversion to open surgery was required in 2 (3.6%) participants. 

**Table 3:** Intraoperative complications and operational time.

Variables	Total number, *n* (%)
Mean surgery time (minutes)	75 ± 15
Intraoperative complications	
Bleeding	3 (5.4%)
Injury to surrounding organs	1 (1.8%)
Conversion to open	2 (3.6%)

Among the immediate postoperative complications, pain was the most frequently reported, affecting 40 (71.4%) participants, followed by nausea or vomiting in 25 (44.6%) participants and infection in 5 (8.9%) participants. Postoperative follow-up rates were recorded at multiple intervals. All participants (56; 100%) were followed up for one week, while 54 (96.4%) attended the one-month follow-up. At three months, 50 (89.3%) participants were followed up, decreasing to 48 (85.7%) at six months and 45 (80.4%) at 12 months. These data highlight the prevalence of immediate complications and the retention of participants over the follow-up period (Table 4).

**Table 4:** Immediate postoperative complications and follow-up.

Variables	Total number, *n* (%)
Immediate postoperative complication	
Pain	40 (71.4%)
Nausea/Vomiting	25 (44.6%)
Infection	5 (8.9%)
Postoperative follow-up (week)	
1 week	56 (100%)
1 month	54 (96.4%)
3 months	50 (89.3%)
6 months	48 (85.7%)
12 months	45 (80.4%)

Among complications, preterm labor was observed in 4 (7.1%) participants, fetal distress in 3 (5.4%), and other complications in 2 (3.6%) participants. Regarding the mode of delivery, 36 (64.3%) participants delivered vaginally, while 20 (35.7%) underwent cesarean delivery. The mean gestational age at delivery was 38.5 ± 1.5 weeks, and the mean birth weight was 3,200 ± 450 g. Neonatal outcomes included a mean APGAR score of 8 ± 1 at one minute and 9 ± 0.5 at five minutes. Notably, there were no cases of fetal death (0; 0%). These data highlights the clinical outcomes for both mothers and neonates in the study (Table 5).

**Table 5:** Obstetric and neonatal outcomes after surgery.

Variables	Total number, *n* (%)
Complications	
Preterm labor	4 (71.%)
Fetal distress	3 (5.4%)
Others	2 (3.6%)
Mode of delivery	
Vaginal	36 (64.3%)
Cesarean	20 (35.7%)
Mean gestational age at delivery (weeks)	38.5 ± 1.5
Mean birth weight (g)	3,200 ± 450
Mean APGAR score at 1 minute	8 ± 1
Mean APGAR score at 5 minutes	9 ± 0.5
Fetal death	0 (0%)

The coefficient for age was 0.07 with a standard error of 0.08, yielding a *P*-value of 0.38, indicating no statistically significant association with immediate postoperative complications. Similarly, the coefficient for BMI is 0.02 with a standard error of 0.11 and a *P*-value of 0.84, suggesting BMI was not a significant predictor. For gestational age, the coefficient was 0.06 with a standard error of 0.059 and a *P*-value of 0.29, indicating no significant predictive value for complications. Parity had a coefficient of 0.33 with a standard error of 0.641 and a *P*-value of 0.60, also showing no significant impact on postoperative outcomes. Finally, the ASA score had a coefficient of 0.17, a standard error of 0.45, and a *P*-value of 0.69, indicating no statistically significant relationship with immediate postoperative complications. The confidence intervals for all variables further reinforce the lack of substantial predictors in our research (Table 6).

**Table 6:** Predictors of immediate postoperative complications (logistic regression analysis). ASA, American Society of Anesthesiologists; BMI, body mass index

Predictors	Coefficient	Standard error	*P*-value	95% confidence interval
Age (years)	0.07	0.08	0.38	-0.088 to 0.229
BMI	0.02	0.11	0.84	-0.19 to 0.237
Gestational age	0.06	0.059	0.29	-0.054 to 0.179
Parity	0.33	0.641	0.60	-0.923 to 1.592
ASA score	0.17	0.45	0.69	-0.708 to 1.063

## Discussion

LC has become the gold standard in managing symptomatic cholelithiasis and chronic cholecystitis, effectively replacing conventional open cholecystectomy [14,15]. The use of laparoscopic techniques in cholecystectomy has expanded rapidly and is now routinely performed in major cities and tertiary care hospitals across the country. While this laparoscopic approach offers numerous advantages, it is also associated with a higher rate of procedure-specific complications, particularly within training institutions [14].

In this study, we aimed to evaluate the outcomes of LC in pregnant women, focusing on both maternal and fetal safety. The increasing prevalence of gallbladder disease among pregnant women underscores the need for careful assessment of surgical interventions during this critical period. The study cohort had a mean age of 32.5 years, with an average BMI of 28.4 kg/m², classifying the participants as overweight. This finding aligns with prior studies indicating that both obesity and advanced maternal age are significant risk factors for gallstone formation during pregnancy [16]. Notably, obesity is a recognized risk factor for gallstone disease, with a direct association between increasing BMI and the incidence of gallstones. This association is consistent with the study by Ko et al. [17], which demonstrated a linear relationship between BMI and gallstone prevalence. Researchers believe that elevated BMI increases susceptibility to cholelithiasis by altering cholesterol metabolism and enhancing cholesterol secretion in bile [18].

Moreover, a substantial proportion of participants in this study (34, 60.7%) were multiparous, which aligns with findings that suggest an elevated risk of gallbladder disease among women with multiple pregnancies [19]. Previous research has documented a correlation between the incidence of gallstones and increasing parity, indicating that the number of pregnancies may serve as a significant risk factor for gallstone formation in women [20].

Comorbid conditions, including diabetes (12, 21.4%) and hypertension (8, 14.3%), were also present in our study cohort, supporting previous findings that link these conditions to an increased risk of biliary tract disease and postoperative complications [21]. In this study, pain was the most frequently reported preoperative symptom, affecting 48 (85.7%) participants, followed by nausea and vomiting in 36 (64.3%) cases. These findings align with the typical presentation of patients undergoing cholecystectomy or similar surgical interventions [14]. Additionally, our results align with existing literature, which indicates that pain and gastrointestinal symptoms, such as nausea and vomiting, are common in cases of symptomatic cholelithiasis, particularly among pregnant individuals [22].

The primary indication for surgery in our cohort was symptomatic cholelithiasis, accounting for 30 (53.6%) cases, followed by cholecystitis at 20 (35.7%) and biliary colic at 6 (10.7%). This distribution is consistent with findings by Nan et al. [23], which similarly identified symptomatic gallstones and acute cholecystitis as the predominant reasons for surgical intervention in pregnant patients. The ASA classification in our study revealed that 30 (53.6%) patients were classified as ASA Class II, indicating mild systemic disease. In comparison, 14 (25%) were Class I, with no systemic disease, and 10 (17.9%) were classified as Class III, reflecting severe systemic disease. This distribution aligns with previous research highlighting the need for ASA classification in assessing patient risk and determining appropriate anesthetic management [20,24].

In terms of intraoperative complications, bleeding was the most frequent issue, occurring in 3 (5.4%) cases, followed by injury to surrounding organs, which occurred in 1 (1.8%) case, and the need for conversion to open surgery, which was required in 2 (3.6%) cases. These complication rates were consistent with those reported in the existing literature, suggesting that laparoscopic techniques can be safely employed without significantly increasing the risk of maternal morbidity [23].

The immediate postoperative complications observed in this study included pain (40, 71.4%) and nausea/vomiting (25, 44.6%), both of which are commonly encountered after surgical procedures. These complications were effectively managed in accordance with our follow-up protocols. Long-term follow-up data indicated that 45 (80.4%) patients remained free of complications 12 months post-surgery, providing evidence for the sustained safety and efficacy of the procedure.

Regarding obstetric outcomes, the rate of preterm labor was 4 (7.1%), consistent with findings reported in the literature. These results suggest that while cholecystectomy during pregnancy is generally considered safe, there remains a slight risk of preterm labor. Importantly, no fetal deaths occurred in our cohort. The average gestational age at delivery was 38.5 weeks, with a mean birth weight of 3,200 g. These outcomes suggest favorable neonatal health following the procedure.

Limitations of the study

This study on LC in pregnant women has several limitations. The absence of a control group restricts the ability to compare outcomes with non-surgical management or other surgical techniques. Additionally, the follow-up period may need to be extended to capture long-term maternal and fetal outcomes, thereby necessitating further research for a more comprehensive understanding of the procedure’s safety and efficacy.

## Conclusions

In conclusion, LC in pregnant women is a feasible and safe procedure with manageable risks for both the mother and fetus. The study demonstrated low rates of intraoperative and postoperative complications, with pain being the most frequently reported postoperative issue. The majority of deliveries were full-term, with favorable neonatal outcomes, and no fetal deaths were reported. Despite the small sample size and retrospective design, the findings support the use of LC as an effective treatment for gallbladder disease during pregnancy, particularly in the second trimester. However, further research with larger cohorts is needed to validate these results and establish comprehensive, evidence-based guidelines.

## References

[1] de Bari O, Wang TY, Liu M, Paik CN, Portincasa P, Wang DQ (2014). Cholesterol cholelithiasis in pregnant women: pathogenesis, prevention and treatment. Ann Hepatol.

[2] Mendez-Sanchez N, Chavez-Tapia NC, Uribe M (2006). Pregnancy and gallbladder disease: symposium on liver & pregnancy. Ann Hepatol.

[3] Sungler P, Heinerman PM, Steiner H (2000). Laparoscopic cholecystectomy and interventional endoscopy for gallstone complications during pregnancy. Surg Endosc.

[4] Liu B, Beral V, Balkwill A (2009). Childbearing, breastfeeding, other reproductive factors and the subsequent risk of hospitalization for gallbladder disease. Int J Epidemiol.

[5] Hedström J, Nilsson J, Andersson R, Andersson B (2017). Changing management of gallstone-related disease in pregnancy - a retrospective cohort analysis. Scand J Gastroenterol.

[6] Soper NJ, Hunter JG, Petrie RH (1992). Laparoscopic cholecystectomy during pregnancy. Surg Endosc.

[7] Hess E, Thumbadoo RP, Thorne E, McNamee K (2021). Gallstones in pregnancy. Br J Hosp Med (Lond).

[8] Othman MO, Stone E, Hashimi M, Parasher G (2012). Conservative management of cholelithiasis and its complications in pregnancy is associated with recurrent symptoms and more emergency department visits. Gastrointest Endosc.

[9] Nasioudis D, Tsilimigras D, Economopoulos KP (2016). Laparoscopic cholecystectomy during pregnancy: a systematic review of 590 patients. Int J Surg.

[10] Sedaghat N, Cao AM, Eslick GD, Cox MR (2017). Laparoscopic versus open cholecystectomy in pregnancy: a systematic review and meta-analysis. Surg Endosc.

[11] Amoli HA, Tavakoli H, Notash AY, Far MS, Khashayar P (2008). Laparoscopic cholecystectomy during pregnancy: a case series. J Minim Access Surg.

[12] Ibiebele I, Schnitzler M, Nippita T, Ford JB (2017). Outcomes of gallstone disease during pregnancy: a population‐based data linkage study. Paediatr Perinat Epidemiol.

[13] Doyle DJ, Hendrix JM, Garmon EH (2023). American Society of Anesthesiologists Classification. https://www.ncbi.nlm.nih.gov/books/NBK441940/.

[14] Cawich SO, Mitchell DI, Newnham MS, Arthurs M (2006). A comparison of open and laparoscopic cholecystectomy done by a surgeon in training. West Indian Med J.

[15] Al Salamah SM (2005). Outcome of laparoscopic cholecystectomy in acute cholecystitis. J Coll Physicians Surg Pak.

[16] Belal S, Hamed HM, Kamal A, Al-Sayed MA, Abd El Hamid HM (2022). Risk factors associated with cholelithiasis during pregnancy and postpartum. Open J Obstetr Gynecol.

[17] Ko CW, Beresford SA, Schulte SJ, Matsumoto AM, Lee SP (2005). Incidence, natural history, and risk factors for biliary sludge and stones during pregnancy. Hepatology.

[18] Moghaddam TG, Fakheri H, Abdi R, bavar Rostami FK, Bari Z (2013). The incidence and outcome of pregnancy-related biliary sludge/stones and potential risk factors. Arch Iran Med.

[19] Hossain GA, Islam SM, Mahmood S, Chakrabarty RK, Akhter N (2003). Gall stone in pregnancy. Mymensingh Med J.

[20] Kolbeinsson HM, Hardardottir H, Birgisson G, Moller PH (2016). Gallstone disease during pregnancy at Landspitali University Hospital 1990-2010. Laeknabladid.

[21] Chen CH, Lin CL, Kao CH (2020). The risk of venous thromboembolism in patients with gallstones. Int J Environ Res Public Health.

[22] Mahjoubi MF, Dhaou AB, Maatouk M, Essid N, Rezgui B, Karoui Y, Moussa MB (2023). Acute cholecystitis in pregnant women: a therapeutic challenge in a developing country center. Ann Hepatobiliary Pancreat Surg.

[23] Nan X, Chan E, Wong KS, Ng J, Izwan S, Cooper M, Damodaran R (2023). Laparoscopic cholecystectomy in pregnancy: a seven-year retrospective study from an Australian tertiary center. Cureus.

[24] Mayhew D, Mendonca V, Murthy BV (2019). A review of ASA physical status - historical perspectives and modern developments. Anaesthesia.

